# Inflammation and Airway Microbiota during Cystic Fibrosis Pulmonary Exacerbations

**DOI:** 10.1371/journal.pone.0062917

**Published:** 2013-04-30

**Authors:** Edith T. Zemanick, J. Kirk Harris, Brandie D. Wagner, Charles E. Robertson, Scott D. Sagel, Mark J. Stevens, Frank J. Accurso, Theresa A. Laguna

**Affiliations:** 1 Department of Pediatrics, University of Colorado School of Medicine, Aurora, Colorado, United States of America; 2 Department of Biostatistics and Informatics, Colorado School of Public Health, University of Colorado School of Medicine, Aurora, Colorado, United States of America; 3 Department of Molecular, Cellular and Developmental Biology, University of Colorado, Boulder, Colorado, United States of America; 4 Department of Pediatrics, University of Minnesota Medical School and the University of Minnesota Amplatz Children’s Hospital, Minneapolis, Minnesota, United States of America; Queens University Belfast, Ireland

## Abstract

**Background:**

Pulmonary exacerbations (PEx), frequently associated with airway infection and inflammation, are the leading cause of morbidity in cystic fibrosis (CF). Molecular microbiologic approaches detect complex microbiota from CF airway samples taken during PEx. The relationship between airway microbiota, inflammation, and lung function during CF PEx is not well understood.

**Objective:**

To determine the relationships between airway microbiota, inflammation, and lung function in CF subjects treated for PEx.

**Methods:**

Expectorated sputum and blood were collected and lung function testing performed in CF subjects during early (0–3d.) and late treatment (>7d.) for PEx. Sputum was analyzed by culture, pyrosequencing of 16S rRNA amplicons, and quantitative PCR for total and specific bacteria. Sputum IL-8 and neutrophil elastase (NE); and circulating C-reactive protein (CRP) were measured.

**Results:**

Thirty-seven sputum samples were collected from 21 CF subjects. At early treatment, lower diversity was associated with high relative abundance (RA) of *Pseudomonas* (r = −0.67, p<0.001), decreased FEV_1%_ predicted (r = 0.49, p = 0.03) and increased CRP (r = −0.58, p = 0.01). In contrast to *Pseudomonas*, obligate and facultative anaerobic genera were associated with less inflammation and higher FEV_1_. With treatment, *Pseudomonas* RA and *P. aeruginosa* by qPCR decreased while anaerobic genera showed marked variability in response. Change in RA of *Prevotella* was associated with more variability in FEV_1_ response to treatment than *Pseudomonas* or *Staphylococcus*.

**Conclusions:**

Anaerobes identified from sputum by sequencing are associated with less inflammation and higher lung function compared to *Pseudomonas* at early exacerbation. CF PEx treatment results in variable changes of anaerobic genera suggesting the need for larger studies particularly of patients without traditional CF pathogens.

## Introduction

Pulmonary exacerbations (PEx), characterized by increased respiratory symptoms and worsening lung function, are the leading cause of morbidity and decreased quality of life in cystic fibrosis (CF) [1]. Recurrent PEx are associated with long term decline in lung function and shortened survival [2]–[4]. The causes of PEx are not fully understood; however, new acquisition or clonal expansion of chronically infecting bacteria, such as *Pseudomonas aeruginosa* and *Staphylococcus aureus,* and the resulting inflammatory response are known to contribute [5]. Treatment typically includes antibiotics targeting airway bacteria; however, airway cultures may not detect bacteria even during exacerbation [6]. In addition, 25% of patients fail to regain baseline lung function after treatment of a PEx, highlighting the need for improved treatment [7].

CF airway infections are frequently polymicrobial [8]–[10]. Given that a quarter of patients fail to fully respond to culture-guided treatment of PEx, improved understanding of airway infection is critical for the development of novel therapeutic approaches. Molecular analyses detect bacteria using polymerase chain reaction (PCR) amplification of bacterial small subunit ribosomal RNA (SSU-rRNA) genes followed by fingerprinting [e.g., terminal restriction fragment length polymorphism (T-RFLP)] or sequencing, most recently with high-throughput pyrosequencing of barcoded SSU- rRNA gene amplicons [11]–[13]. Using strict anaerobic culture techniques and these molecular approaches, obligate and facultative anaerobes have been detected in high quantities from CF sputum during PEx. However, the contribution of anaerobes to PEx and lung disease in CF is incompletely understood [14]–[17]. Ecologic characteristics of the microbiota (e.g. diversity) have also been associated with lower lung function in CF, suggesting their potential use as a biomarker of lung disease [18].

Based on these studies, we hypothesized that airway microbial diversity and abundance of obligate and facultative anaerobes would be associated with increased airway inflammation and decreased lung function during CF PEx. In order to test this hypothesis, we sought to ascertain the relationship between airway microbiota, biomarkers of airway and systemic inflammation, and lung function in subjects with CF early in treatment of a PEx. In addition, we sought to determine changes in the airway microbiota following at least one week of intravenous (IV) antibiotic therapy, and to correlate these shifts with changes in inflammation and lung function.

## Methods

### Ethics Statement

The study was approved by the Colorado Multiple Institutional Review Board (COMIRB). Written informed consent and HIPPA Authorization were obtained from all participants over the age of 17 years or from parents or legal guardians of participants younger than 18 years. Assent was obtained from all participants under 18 years.

### Study Population and Design

Patients with a diagnosis of CF (sweat chloride >60 mEq/L and/or two known CF mutations) and a clinically diagnosed PEx requiring hospitalization were recruited at the time of admission. This study was designed to measure changes in desmosine, a biomarker of lung injury; details of this study were previously published [19], [20]. Our analysis included a cohort of subjects from the original study who were able to provide expectorated sputum on admission; preliminary data was published as an abstract previously [21].

Subjects were evaluated at two time points during hospitalization, *early treatment* (day 0–3) and following ≥7 days of IV antibiotics or *late treatment* (day 7–14). These time points were chosen because most lung function improvement during PEx treatment is seen within this time period [22], [23]. Study evaluation consisted of spirometry and collection of expectorated sputum and blood specimens. Spirometry was performed according to American Thoracic Society guidelines [24]. Antibiotics administered during hospitalization, at the discretion of the attending physician, were recorded.

#### Sputum collection, processing and microbiologic analyses

Detailed descriptions of sputum collection and processing are provided in Data S1. Culture was performed on the early treatment sputum specimen following standard guidelines [25]. Total and specific bacterial qPCR assays were used to measure *P. aeruginosa, Prevotella denticola, Prevotella melaninogenica* and *Prevotella oris;* details of the qPCR assays are provided in the Data S1 and have been published; primers are shown in Table S1 [26], [27].

### Biomarkers of Inflammation

Sputum samples were analyzed as previously described for cytology, interleukin-8 (IL-8) [Luminex Multiplex Bead, R&D Systems; Abingdon, Oxon, UK] and free neutrophil elastase (NE) activity [Spectrophotometric assay, Sigma Diagnostics; St. Louis, MO] [19]. Plasma C-reactive protein (CRP; Cardiophase hsCRP, BN 11 instrument; Siemens; Deerfield, IL) was measured from blood samples (EDTA Plasma). Additional details of these assays are provided in the Data S1.

#### DNA extraction

DNA extractions were performed using the Qiagen EZ1 Advanced automated extraction platform (Qiagen Inc., Valencia, CA) with the bacterial card and tissue extraction kit. All sample manipulation was done in the BSL2 hood with appropriate laminar flow. Homogenized frozen sputum samples were thawed at 4°C and vortexed to ensure mixing. An aliquot of 200 µl for extraction was transferred into the tube provided with the EZ1 kit. Remaining sample was placed in a clean 2 ml tube and stored at −80°C. Extraction reagent cartridges, elution tubes and tip holders were loaded into the EZ1 sample rack as instructed by the manufacturer. Elution volume of 100 µl was selected and EZ1 DNA Tissue Kit program was run. Elution tubes with DNA extract were stored at −20°C.

#### DNA sequencing

Pyrosequencing of barcoded 16S rRNA gene amplicons was used to determine the bacterial communities present in each sample. We have established protocols for the efficient construction of highly multiplexed amplicon pools that exploit barcoded PCR primers (Figure S1) to allow assignment of each sequence detected to the appropriate sample [11], [28]. Each DNA extract was PCR amplified in triplicate with a specific barcoded primer. A negative amplification control was run for all barcodes, and any sample where the control exhibited amplification by agarose gel electrophoresis underwent repeat PCR amplification. PCR reactions consisted of 10 µl 2.5X HotMaster PCR Mix (Eppendorf), 0.3 µM each primer, 10–100 ng template DNA in a total reaction volume of 25 µl. PCR reactions were cycled with an Eppendorf Mastercycler using the following cycling parameters: 2 minutes denaturating at 95°C followed by 30 cycles of 20 s at 95°C (denaturing), 20 s at 52°C (annealing) and 60 s at 65°C (elongation). Mixtures were normalized using the SequalPrep Kit (Invitrogen) and mixed in equal volumes [28]. The pooled amplicons were concentrated by evaporation and gel purified (Montage) prior to sequencing. Barcoded libraries of rRNA amplicons were sequenced using the 454 platform (454 Roche Life Sciences, Branford, CT) [11]. The primers (27F/338R) target the V1/2 region of the bacterial rRNA gene. The linker sequence included in our fusion primer is a 2-nucleotide feature that disrupts hybridization between the rRNA primer and barcode/454 adaptor. This feature was designed by changing the last 2 bases in the rRNA primer, and was added due to lineage specific sequence conservation within the barcode or adaptor sequences that may have favored particular lineages in a primer specific manner [11]. The DNA sequencing data was deposited in the NCBI Short Read Archive database under the accession SRA052781 following guidelines from the National Center for Biotechnology Information [29].

#### Post sequencing informatics

Initial quality checks were performed during the de-convolution of the amplicon pool based on the primer barcode sequence using the software package BARTAB [30]. These checks included identification of a legitimate barcode sequence, sequencing length (>200 nucleotides, >Q20 using a 10 base sliding window), and excluding ambiguous bases. Additional quality checks consisted of alignment with Infernal to confirm that the sequences conformed to the bacterial rRNA secondary structure and ChimeraSlayer to detect sequences derived from multiple templates and exclude chimeras [31], [32]. RDP Classifier was used to identify the source of all sequences that pass these quality checks. A standalone version of the RDP classifier java code version 2.4 with MSU training set V7 (dating from Jan 12, 2012) was used [33], [34]. Only rank assignments with bootstrap confidence estimates greater than 50% were retained in the RDP Classifier results [35]. We classified bacterial genera as aerobic to facultative anaerobe or obligate anaerobe based on previous publications in CF as shown in Table 1, although we recognize that there is some overlap in phenotypic behavior between these groups [13]–[16], [38].

**Table 1:** Classification of the most prevalent bacterial genera detected from CF sputum samples.

Aerobic to facultative anaerobic genera	Obligate anaerobic genera
*Atopobium*	*Actinomyces*
*Capnocytophaga*	*Campylobacter*
*Granulicatella*	*Fusobacterium*
*Haemophilus* *	*Leptotrichia*
*Lactobacillus*	*Porphyromonas*
*Pseudomonas* *	*Prevotella*
*Rothia*	*Veillonella*
*Staphylococcus* *	
*Stenotrophomonas* *	
*Streptococcus*	

* Indicates genus that includes bacterial species typically detected by standard CF microbiologic culture.

We also performed an ancillary analysis using BLAST to compare study sequences versus all of the isolate sequences in Silva Release 104 labeled as “isolate” to attempt a species level assignment for each sequence [36], [37]. BLAST hits were required to have E values of at least 10^−5^ and to overlap the Silva isolate sequence by at least 95%. The Silva taxonomy line associated with the best BLAST hit was required to match the taxonomy line reported by RDP at the genus level in order to append the binomial name of the isolate to the RDP taxonomy line [36]. For select genera of particular interest, specifically, *Pseudomonas, Staphylococcus, Haemophilus, Stenotrophomonas, Prevotella* and *Streptococcus,* species names were examined. Counts for each unique taxonomic line were tabulated for each sample, and tables containing all samples in an analysis were generated. Independent tables were generated for RDP and the BLAST extended analysis.

We used classification approaches rather than numerical operational taxonomic units (OTUs) based on pairwise sequence comparisons for several reasons. First, the classifier assignments are stable (within a software release and training set) unlike OTUs where the sample composition of the analysis affects the outcome. Second, each sequence is independently classified even within a sample. This eliminates the optimization across all sequences that are required for numerical OTUs, which is highly dependent on the specific algorithm used for OTU selection. Finally, the RDP classification approach generates a bootstrap confidence estimate for each level of taxonomic assignment that can be used to filter the results further (e.g. analysis of only sequences that are identified to genus level).

### Statistical Analysis

Subject variables were described using percentages, median and range values. We performed a cross-sectional analysis of all subjects who had a sputum sample collected at early treatment of PEx, and a paired-sample analysis for a subgroup of subjects who had sputum samples collected at both early and late treatment PEx. Inflammatory biomarkers were log_10_ transformed and anchored at 1. Total and specific bacterial loads were determined by qPCR in triplicate; these values were log_10_ transformed, anchored at 1 and then averaged. Change in qPCR measurements with treatment were described using the median and range values and evaluated using a Signed rank test. Change in FEV_1%_ predicted, inflammatory biomarkers and qPCR with treatment were described using the median and range values and evaluated using a Signed rank test.

Pyrosequencing: Shannon Diversity, an ecologic parameter describing the richness and evenness of a microbial community, was determined for each sample using rarefaction to the number of sequences in the smallest library [38]. The number of effective species, or the true diversity, was also calculated [39]. This measure aids in the interpretation and comparison of the diversity indices by transforming the number into a variable that corresponds to the number of equally-common species. To account for variable sequencing effort, relative abundance (RA) of each genus was calculated by dividing the sequence counts by the total number of sequences, and used for further analyses. This approach takes into account that the differences in the total number of sequences for each case can vary and transforms the pyrosequencing information into relative quantities rather than absolute sequence counts. Genera detected at early treatment were described using median and range values as well as the proportion of samples for which the genera was detected. The genera were ranked by RA within each sample and the most dominant (top ranked) genus was identified. Principal Component Analysis (PCA) was performed on sputum microbial and inflammatory markers using the correlation matrix after transforming the microbial measurements using the centered log ratio transformation recommended for compositional data [31], [32]. The resulting principal components, linear combinations of the microbial and inflammatory markers, were correlated with FEV_1_% predicted using a Spearman’s rank correlation coefficient. The associations between bacterial genera, clinical parameters and inflammatory biomarkers at the early treatment visit were also evaluated using Spearman’s rank correlation coefficient, and corresponding confidence intervals were calculated using Fisher’s transformation.

Subjects who had sputum samples collected at both early and late treatment PEx were included in a paired-sample analysis. Change in airway microbiology was plotted using the median of samples where the genus was detected in either early or late treatment. Particular genera of interest were analyzed further using a Signed rank test. To examine the relationship of change in bacterial genera with changes in inflammation and change in FEV_1_% predicted, the differences between early and late treatment measurements were calculated. The association between these differences was evaluated using Spearman’s rank correlation coefficients. Confidence intervals for these correlations were estimated using Fisher’s transformation. To estimate the contribution of changes in specific bacterial genera in explaining the variability in response to treatment, defined as the change in FEV_1_% predicted, a linear regression model was used to obtain R^2^ values and corresponding confidence intervals [40]. Comparison of pyrosequencing to culture results was performed by calculating sensitivity and specificity using culture as the gold standard. For all analyses, p-values ≤0.05 were considered significant, and p-values of 0.06 to 0.1 were considered marginally associated. Analyses were performed using SAS Version 9.2 software (SAS Institute Inc.: Cary, NC, 2008).

## Results

### Subject Characteristics and Specimens Collected

Fifty-three CF subjects were recruited for the study. Twenty-one subjects were able to spontaneously expectorate sputum within three days of admission and were included in this analysis. Subject characteristics are shown in Table 2. Thirty-seven sputum samples were obtained consisting of 21 early treatment and 16 paired late treatment samples. One sputum sample did not have sufficient quantity for inflammatory analyses. As not all subjects had a late treatment sputum sample (due to discharge home at <7 days in two subjects, inability to spontaneously expectorate in two subjects, and missing sputum sample in one), we examined our data in two ways: (1) cross-sectional analysis of 21 subjects at early treatment of PEx, and (2) paired analysis of 16 subjects with both early and late treatment PEx sputum samples. Subjects with paired early and late treatment samples showed significant improvement in FEV_1_ and decreases in airway inflammatory markers, IL-8 and NE, and circulating CRP in response to PEx treatment (Table 2). Inflammatory and FEV_1_ changes with treatment were similar to that reported in previously published studies of CF PEx [41], [42]. Subject characteristics, microbiologic data, lung function, sputum NE, CRP and antibiotic treatment for individual subjects are provided in Table S2.

**Table 2:** Subject Characteristics.

	All Subjects (n = 21)	Subjects with paired late treatment data (n = 16)
Female, n (%)	11 (53)	9 (56)
Length of stay, days, median (range)	10 (4–21)	12 (9–21)
Age, yr, median (range)	19 (6–37)	20 (12–37)
FEV_1%_ predicted, median (range)	43 (26–107)	44 (26–107)
Day of sputum collection, median (range)	2 (0–3)*	9 (7–11)†
Genotype, n (%)		
ΔF508/ΔF508	9 (43)	8 (50)
ΔF508/Other	9 (43)	5 (31)
Other/Other	3 (14)	3 (19)
Sputum Culture Results, n (%):		
>1 pathogen	17 (81)	13 (81)
* Pseudomonas aeruginosa*	14 (67)	12 (75)
* Methicillin-sensitive S. aureus*	7 (33)	4 (25)
* Methicillin-resistant S. aureus*	6 (29)	4 (25)
* Stenotrophomonas maltophilia*	1 (5)	0 (0)
AFB positive	2 (10)	2 (13)
Fungi‡	6 (29)	3 (19)
Change with treatment, median (range) in:		
FEV_1%_ predicted		12 (−7 to 34)§
Sputum IL-8, log pg/mL		−0.4 (−1.6 to 0.4)§
Sputum NE, log µg/mL		−0.5 (−1.4 to 0.5)§
CRP, log mg/L		−0.06 (−0.7 to 0.02)§
IV Antibiotics, n (%)		
Tobramycin	17 (81)	
Cefipime	11 (52)	
Ceftazidime	3 (14)	
Meropenem	7 (33)	
Imipenem	1 (5)	
Piperacillin-tazobactam	1 (5)	
Vancomycin	2 (10)	
Other (Amikacin, Aztreonam)	2 (10)	
Oral and inhaled antibiotics, n (%)		
Tobramycin, inhaled	4 (19)	
Aztreonam, inhaled	3 (14)	
Fluoroquinolone	6 (29)	
Linezolid	4 (19)	
Doxycycline	4 (19)	
Trimethoprim-sulfamethoxazole	3 (14)	
Antifungal	7 (33)	
Chronic azithromycin	18 (86)	
Other	5 (24)	

* Early treatment sputum samples;

† Late treatment sputum samples;

‡ Fungi consisted of *Aspergillus fumigatus* (3), *Scedosporium apiospermum* (2), *Scedosporium* sp. (1), *Aspergillus terreus* (1) and *Penicillum* (1);

§ p-value <0.01.

### Microbiota in Early Treatment of PEx: Cross-sectional Sputum Results

#### Pyrosequencing

**Figure 1:**
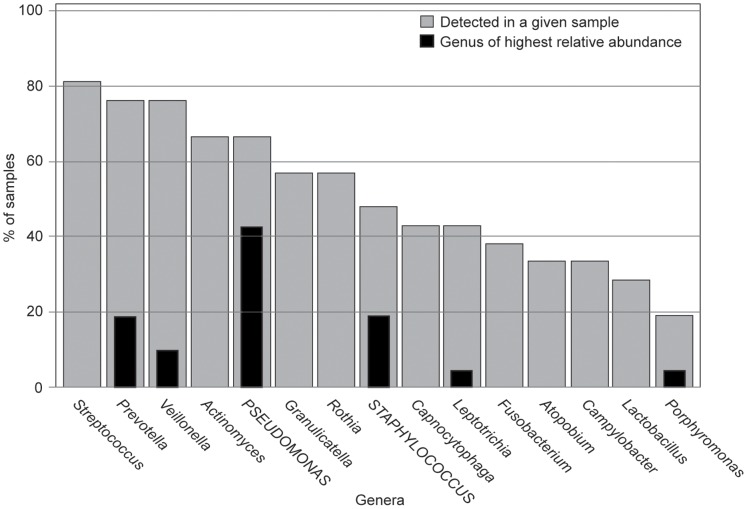
Summary of most prevalent and top ranked (highest relative abundance) genera from early treatment CF sputum samples. Prevalence of genera that are top ranked or detected in more than 25% of early-treatment sputum samples are shown. Grey bars show the percent of samples with a given bacterial genera detected by pyrosequencing. Black bars indicate the percent of samples in which the bacterial genera was the top ranked genus detected. (N = 21 early treatment sputum samples).

There were 75 bacterial genera detected from the 21 early treatment sputum samples with a median of 12 genera per sample (range, 2–29). The total number of sequences per sample varied from 195–2,644 with a median count of 809 sequences per sample; relative abundance (RA) of each genus was calculated and used for further analysis. *Pseudomonas* or *Staphylococcus* was the top ranked genus detected in 62% of subjects; an obligate anaerobe was top ranked in the remaining 38% (Table 3). The prevalence of the most frequently detected and top ranked genera are shown in Figure 1. All sputum samples had at least one CF-associated genera detected by pyrosequencing, *Pseudomonas* (67%), *Staphylococcus* (48%), *Haemophilus* (10%), and *Stenotrophomonas* (5%). No samples contained the genus *Burkholderia* by either culture or pyrosequencing. A list of genera detected from all sputum samples (early and late treatment) in greater than 1% relative abundance are listed in Table S3. The most common species within the genera *Pseudomonas*, *Staphylococcus*, *Haemophilus*, *Stenotrophomonas*, *Prevotella* and *Streptococcus* were determined using BLAST (Data S1 and Figure S2). Because of previous reports suggesting that *Streptococcus milleri* group may contribute to CF PEx, we interrogated our data for sequences consistent with the *S. milleri* group (*S. anginosus, S. intermedius,* and *S. constellatus*) [43]. Five of twenty-one subjects (24%) had either *S. anginosus* or *S. intermedius* detected; however, only one subject had *S. milleri* group detected at >1% RA (1% *S. anginosus* and 4% *S. intermedius*).

**Table 3:** Top ranked genus identified in early treatment sputum samples.

Genus	Specimens with genus top ranked, n (%)	% Relative abundance, median (range)
*Pseudomonas*	9 (43)	91 (53–99)
*Staphylococcus*	4 (19)	52 (45–79)
*Prevotella*	4 (19)	49 (35–79)
*Veillonella*	2 (10)	35 (28–43)
*Leptotrichia*	1 (5)	37 (n/a)
*Porphyromonas*	1 (5)	66 (n/a)

N = 21 specimens.

#### Quantitative PCR assays

The bacterial load determined by our qPCR assay detected bacterial DNA in 100% of early treatment sputum samples with a mean quantity (SD) of 9.8 (0.8) log_10_ rRNA gene copies/mL. *P. aeruginosa* was detected in 81% of samples with a mean quantity (SD) of 6.9 (3.7) log_10_ rRNA gene copies/mL. Additional results for specific bacterial qPCR assays are available in Table S4.

### Cross-sectional Relationship between Airway Microbiota, Lung Function and Inflammation in Early Treatment of PEx

**Figure 2:**
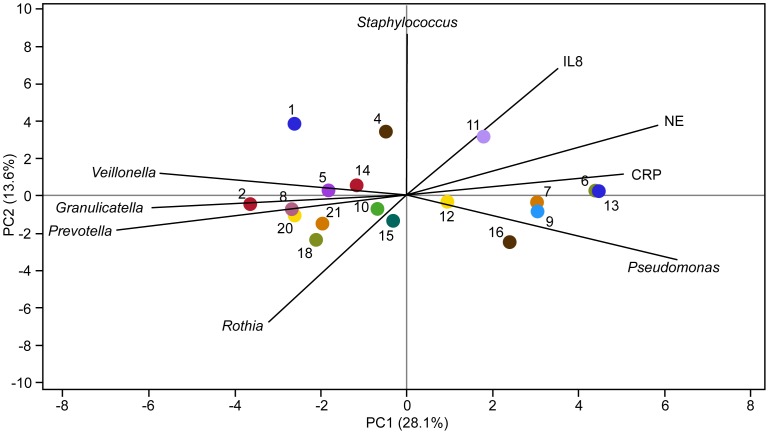
PCA plot of bacterial genera relative abundance and inflammatory markers at Early Treatment of PEx. Colored dots represent individual subjects with subject identification number (SID) (n = 21). The length of the vectors represents the PCA loadings of the variables on the first two principal components, which explain 42% of the variability. *Staphylococcus* and *Pseudomonas* are both positively correlated with NE and CRP, while *Veillonella*, *Granulicatella*, *Prevotella* and *Rothia* are negatively correlated.. The first principal component (PC) was negatively correlated with FEV_1%_ predicted indicating that sputum samples with higher inflammation and higher RA of *Pseudomonas* had lower FEV_1_ and those with lower inflammation and higher RA of *Veillonella*, *Granulicatella* or *Prevotella* had higher FEV_1_.

**Figure 3:**
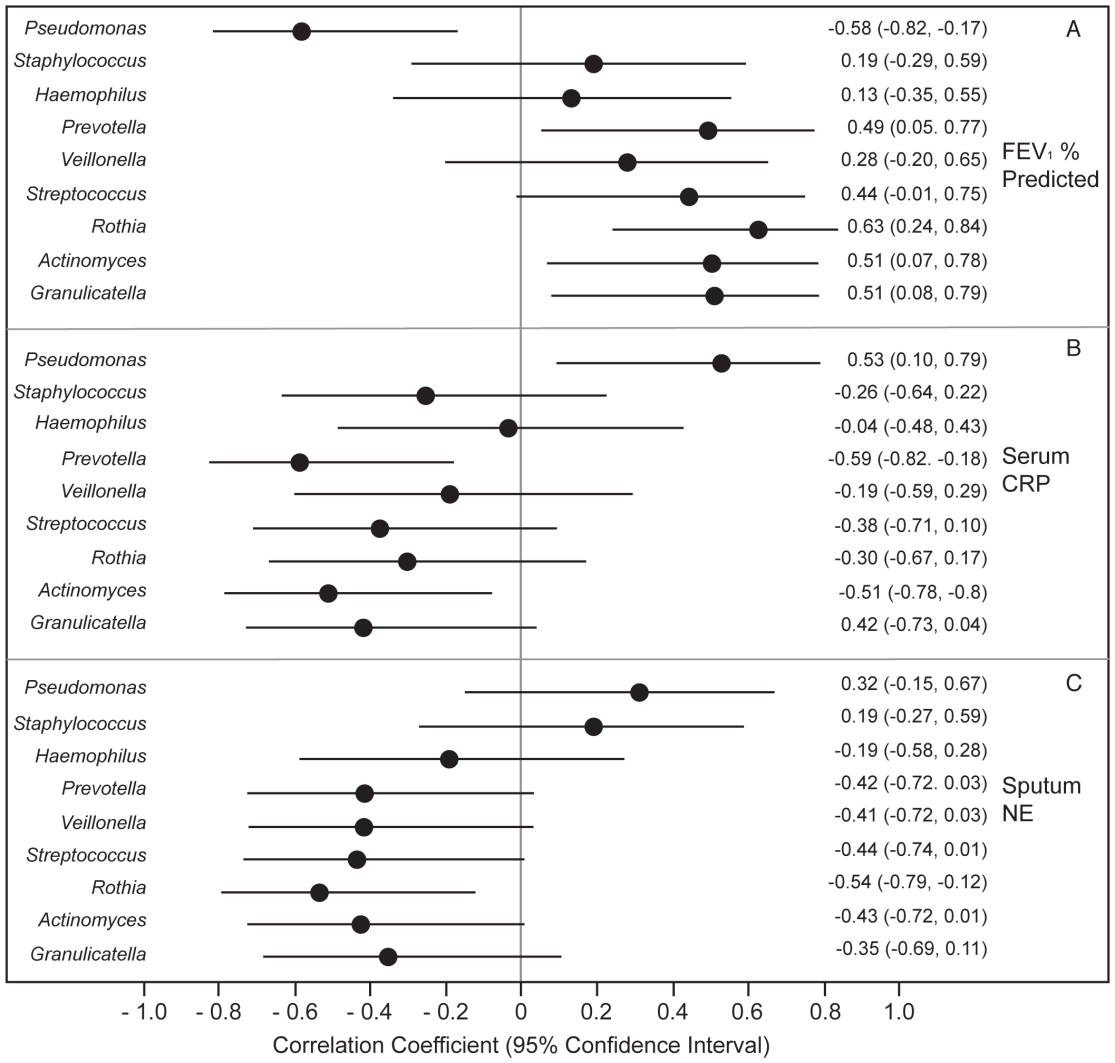
Cross-sectional relationship between airway microbiota, lung function and inflammation measured at Early Treatment of PEx. (n = 21 subjects) Spearman’s rank correlation coefficients and 95% confidence intervals (bars) are shown, measuring the association between the relative abundance of each genera with FEV_1_% predicted, C-reactive protein (CRP) and sputum neutrophil elastase (NE).

We examined the relationship between RA of particular genera, lung function and inflammatory biomarkers at early treatment using PCA (Figure 2) and Spearman’s rank correlation coefficient (Figure 3). Using PCA, *Staphylococcus* and *Pseudomonas* both were positively correlated with sputum NE and circulating CRP, while *Veillonella*, *Granulicatella* and *Prevotella* were negatively correlated. The first principal component (PC) was negatively correlated with FEV_1%_ predicted (r = −0.71, p<0.01), indicating that sputum samples with higher inflammation and higher RA of *Pseudomonas* had lower FEV_1_ and those with lower inflammation and higher RA of *Veillonella*, *Granulicatella* or *Prevotella* had higher FEV_1_. This PC was not associated with age (r = 0.32, p = 0.20). Similarly, using correlation analysis, *Pseudomonas* RA was associated with lower FEV_1_ and increased CRP; *Streptococcus* and the most prevalent obligate anaerobes, *Prevotella, Veillonella* and *Actinomyces* had the opposite associations, although not all were statistically significant. (Table 4) Other facultative and obligate anaerobes including *Granulicatella, Rothia, Leptotrichia, Porphyromonas,* and *Lactobacillus* had similar patterns with either no or weakly positive associations with FEV_1_and negative associations with inflammatory markers.

**Table 4:** Association between RA of particular genera detected by sequencing, FEV_1_, age and inflammatory markers at early treatment (n = 21 sputum samples).

Genus	FEV1% Predicted	Age	Sputum neutrophil elastase	Sputum IL-8	Serum CRP
	SCC (p-value)	SCC (p-value)	SCC (p-value)	SCC (p-value)	SCC (p-value)
*Actinomyces*	0.51 (0.02)*	−0.05 (0.84)	−0.43 (0.05)*	−0.21 (0.36)	−0.51 (0.02)*
*Pseudomonas*	−0.58 (0.01)*	0.66 (<0.01)*	0.32 (0.17)	0.12 (0.61)	0.53 (0.02)*
*Staphylococcus*	0.19 (0.42)	−0.5 (0.02)*	0.19 (0.41)	0.42 (0.06)	−0.26 (0.28)
*Prevotella*	0.49 (0.03)*	−0.32 (0.15)	−0.42 (0.06)	−0.37 (0.1)	−0.59 (0.01)*
*Veillonella*	0.28 (0.23)	0.07 (0.76)	−0.41 (0.06)	−0.29 (0.2)	−0.19 (0.43)
*Granulicatella*	0.51 (0.02)*	−0.13 (0.56)	−0.35 (0.12)	−0.08 (0.74)	−0.42 (0.07)
*Rothia*	0.63 (<0.01)*	−0.33 (0.14)	−0.54 (0.01)*	−0.32 (0.16)	−0.3 (0.2)
*Leptotrichia*	0.06 (0.8)	0.15 (0.52)	−0.02 (0.93)	0.11 (0.65)	−0.19 (0.44)
*Porphyromonas*	0.01 (0.97)	−0.46 (0.03)*	0.001 (0.99)	−0.29 (0.2)	−0.02 (0.95)
*Lactobacillus*	0.19 (0.44)	0.15 (0.51)	−0.05 (0.82)	−0.16 (0.49)	−0.3 (0.21)

SCC: Spearman’s Correlation Coefficient;

* indicates P-values <0.05.

**Figure 4:**
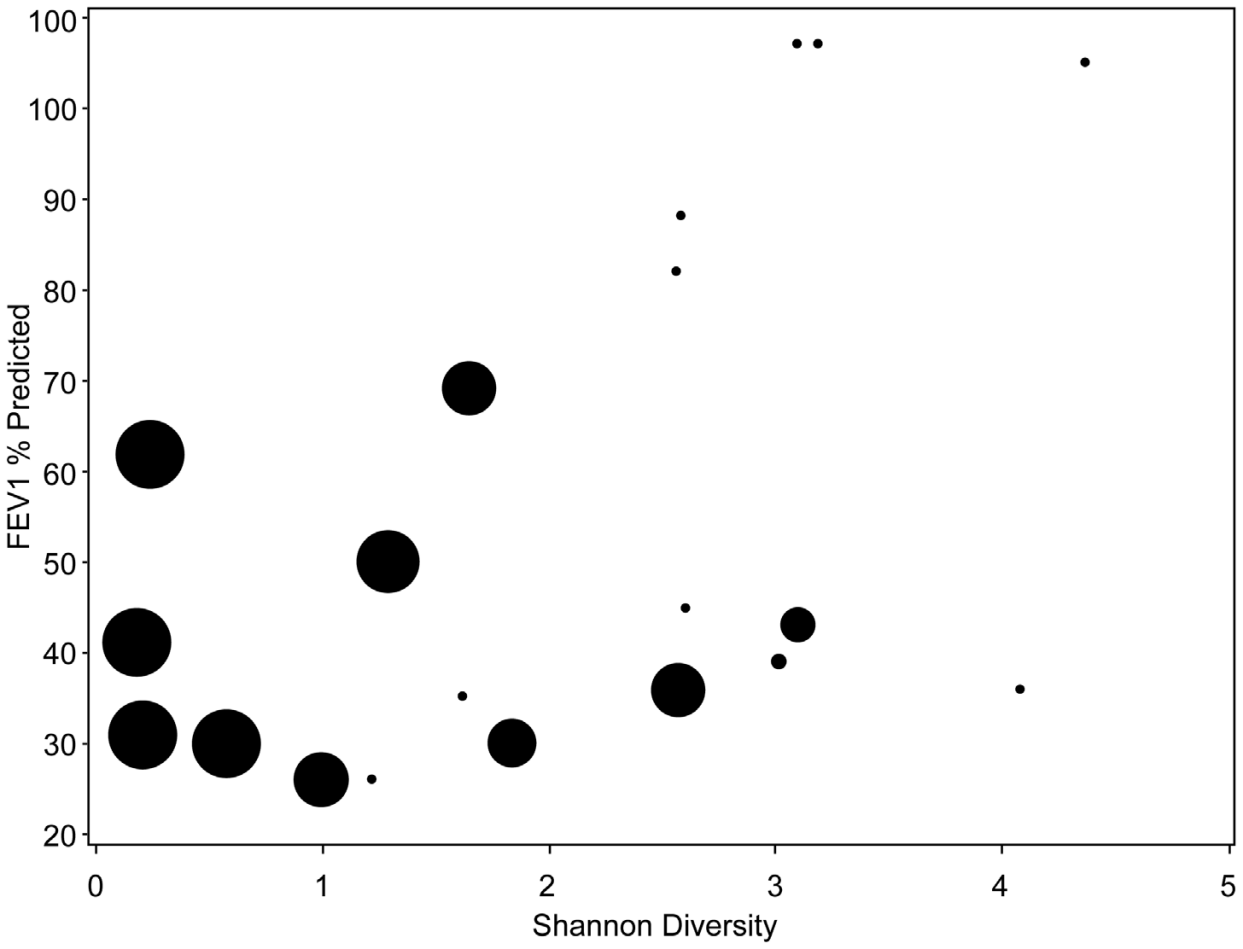
Relationship between diversity, FEV_1_ and *Pseudomonas* RA. Lower diversity is associated with lower FEV_1_ percent predicted and higher RA of *Pseudomonas*. Circle size corresponds to relative abundance of *Pseudomonas* demonstrating that samples with larger relative abundance of *Pseudomonas* have lower diversity, while samples with no or low relative abundance of *Pseudomonas* (small circles) generally have higher diversity.

We also examined the relationship between diversity, *Pseudomonas* RA, inflammation and lung function. (Figure 4) At early treatment, lower diversity was associated with higher RA of *Pseudomonas* (r = −0.68, p = 0.0006), lower FEV_1_ percent predicted (r = 0.49, p = 0.03) and increased CRP (r = −0.58, p = 0.01) and marginally associated with sputum NE (r = −0.43, p = 0.06). *Pseudomonas* RA, but not diversity, was associated with older age (r = 0.7, p = <0.001 versus r = −0.22, p = 0.33 for *Pseudomonas* RA and diversity, respectively). Total bacterial load and specific bacteria measured at early treatment by qPCR were generally not as strongly correlated with lung function and inflammatory markers. However, similar to sequencing data, *P. aeruginosa* tended to have positive correlations with inflammatory markers and negative correlation with FEV_1_ (FEV_1_ correlation with *P. aeruginosa*, r = −0.41, p = 0.08), while correlations with *Prevotella* species went in the opposite direction (Table S5).

### Change in Airway Microbiology with Treatment of a PEx: Paired-sample Results

We examined changes in airway microbiology by comparing paired early and late treatment sputum samples (n = 16).

#### Pyrosequencing

**Figure 5:**
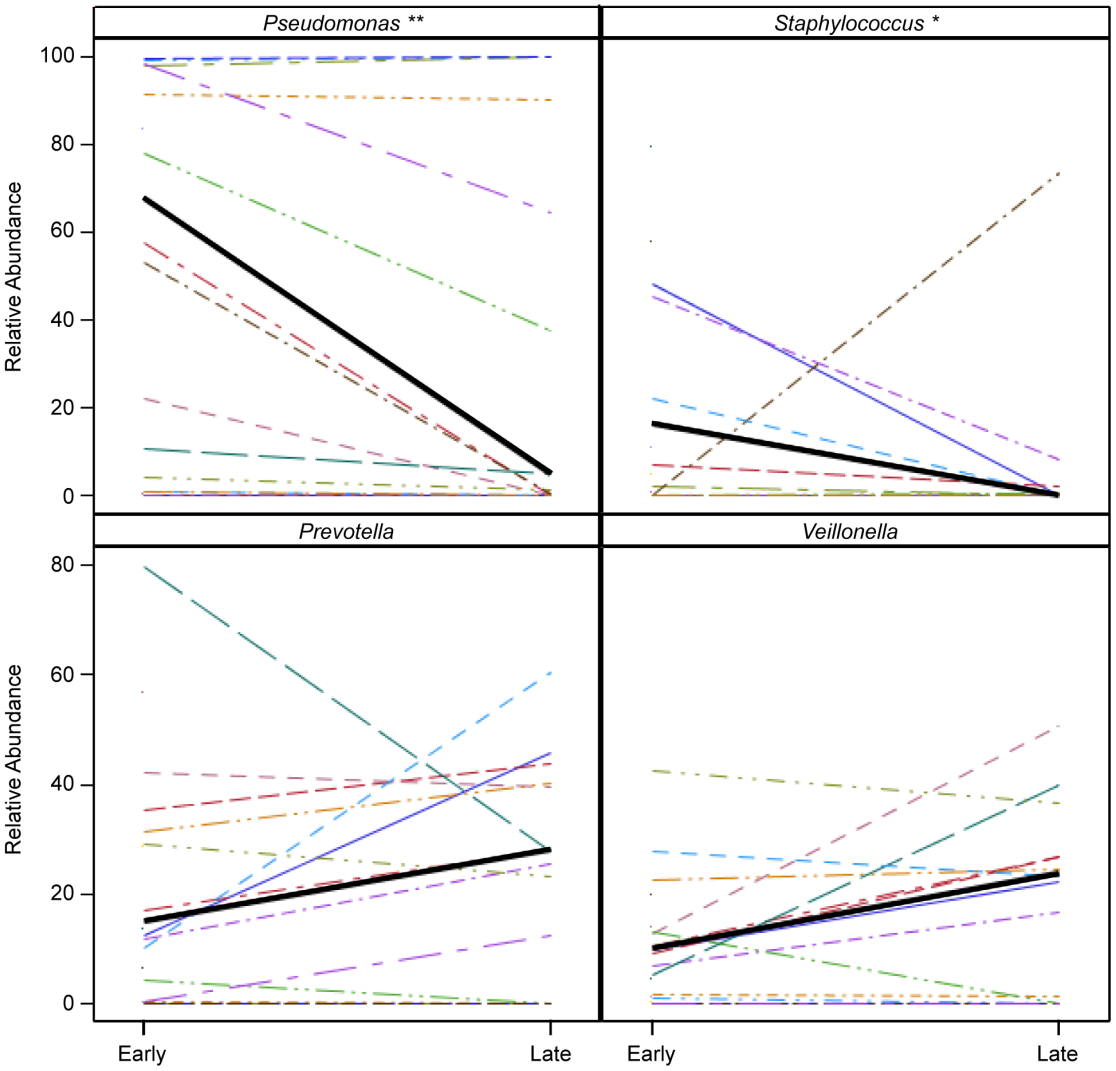
Bacterial genera with the largest magnitude of change in relative abundance by pyrosequencing with treatment of PEx. The median values for samples where the genus was detected during early treatment are connected with a thick black line. Statistically significant and marginally significant differences are represented by **(p = 0.01) and *(p = 0.06), for *Pseudomonas* and *Staphylococcus* respectively. (N = 16 subjects).

Diversity did not change significantly between paired samples [median change (IQR) 0.06 (1.1), p = 0.80], although some individual subjects experienced marked increases or decreases in diversity and effective number of species (Table S2). There were no associations between change in diversity or total bacterial load and RA of *Pseudomonas*, FEV_1_, age or genotype. Changes with treatment in the RA of *Pseudomonas*, *Staphylococcus*, *Prevotella*, and *Veillonella* are displayed in Figure 5; only *Pseudomonas* RA decreased significantly (p = 0.01) while *Staphylococcus* RA trended down (p = 0.06). *Leptotrichia* RA, a known oral genus with pathogenic potential, also trended down with a decrease in four subjects, possibly reflecting its relative sensitivity to cephalosporins (p = 0.08) [44]. We considered whether the lack of change in diversity or *Pseudomonas* RA in some subjects was due to sample collection after the start of IV antibiotics. We examined the relationship between diversity, *Pseudomonas* RA, and change in these parameters with day of collection for early treatment samples (day 0–3) and found no correlations, suggesting that date of collection does not fully explain the lack of change in diversity or *Pseudomonas* RA (Figure S3). The bacterial communities detected during early and late treatment are shown in Figure S4.

#### Quantitative PCR assays

Bacterial load did not change significantly between paired early and late treatment samples [median change (range) −0.12 (−2.5 to 1.6) log_10_ gene copies/mL, p = 0.4]. By qPCR, *P. aeruginosa* decreased in both prevalence and quantity [81% versus 44% positive; median change (range) −3.0 (−10.0 to 0.03) log_10_ gene copies/mL, p<0.01]. *Prevotella* species did not change significantly with treatment, although the prevalence of *P. melaninogenica* and *P. oris* was lower at late treatment and *P. oris* trended down (p = 0.07) (Table S4).

### Relationship between Change in Microbiota, Inflammation and Lung Function with Treatment of PEx

We examined the association between changes in individual genera with changes in lung function and inflammatory markers (Figure S5 and S6). Decreased *Pseudomonas* RA was associated with improvements in FEV_1_ percent predicted (r = −0.5, p = 0.05), but not with changes in inflammatory markers (p-values >0.3). Change in *Prevotella* RA was marginally associated with FEV_1%_ predicted (r = −0.48, p = 0.06) and sputum NE (r = −0.5, p = 0.07) although change in lung function was in the same direction as inflammatory markers. When examined closely, these associations were driven by three subjects with >15% change in *Prevotella* RA. Two subjects with large increases in *Prevotella* (33% and 50% increase in RA) experienced a decrease in FEV_1,_ while one subject with a decrease in *Prevotella* of 50% had improvement in FEV_1_ of 30% predicted.

**Figure 6:**
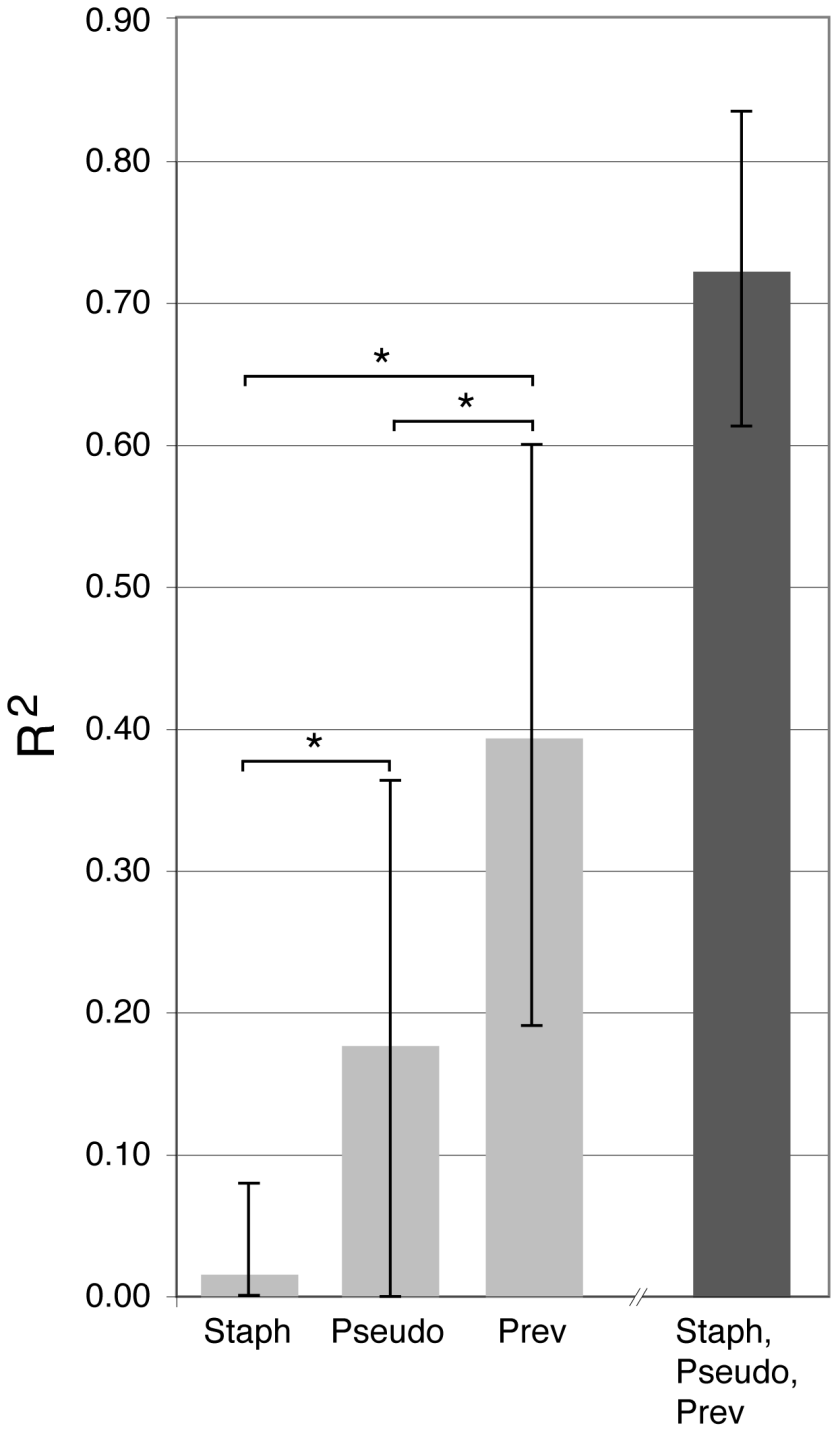
Contribution of change in relative abundance of the genera *Staphylococcus* (Staph), *Pseudomonas* (Pseudo), and *Prevotella* (Prev) and all three combined in explaining the variation in response to treatment measured by change in FEV_1%_ predicted. The height of the bars correspond to the R-squared values, the lines indicate the upper 95% confidence intervals. The black bar corresponds to a model with all three predictors while the grey bars contain one predictor; R-squared values should only be compared across models with the same number of predictors. *Indicates p-values less than 0.05.

Other anaerobic genera including *Veillonella* and *Porphyromonas* were not associated with change in FEV_1_ or inflammatory markers, with the exception of *Leptotrichia* which was negatively associated with change in CRP. Individual patient information including antibiotic treatment is available in Table S2. We also examined the utility of change in genera as a marker of treatment response using a linear regression model. Change in RA of *Pseudomonas* and *Staphylococcus* explained 18% and 2% of the variability of change in FEV_1_ respectively, whereas change in *Prevotella* explained 39%. In a separate model that contained all three genera, 72% of the variability in FEV_1_ change was explained (Figure 6 and Table S6). In this model, *Prevotella* had the largest F-statistic of the three genera, thus, changes in *Prevotella*, after controlling for *Pseudomonas* and *Staphylococcus*, helped explain much of the variability in FEV_1_ response to treatment.

### Comparison of Pyrosequencing and qPCR to Culture for Detection of CF Pathogens

Data comparing pyrosequencing and specific bacterial qPCR assays to culture for CF pathogens are available in Data S1 and Figure S7.

## Discussion

Using molecular sequencing for bacterial identification, we found that obligate and facultative anaerobic genera detected from expectorated sputum samples early in treatment of CF PEx were associated with lower inflammation and higher lung function whereas *Pseudomonas* RA was associated with increased inflammation and lower lung function. Change in *Pseudomonas* RA with treatment was associated with improvement in lung function, whereas change in anaerobic genera was variable and was not associated with FEV_1_ response to treatment, possibly due to the use of anti-pseudomonal antibiotics with varying anaerobic activity. The exception among obligate anaerobic genera was *Prevotella,* which was marginally associated with change in lung function, but was not associated with changes in CRP or sputum inflammatory markers. All subjects had at least one CF pathogen detected during early treatment; thus, our findings may not be applicable to a patient group with negative sputum cultures [6]. In addition, we relied on spontaneously expectorated sputum samples, suggesting that patients included in this cohort may have more significant lung disease compared to non-expectorating patients.

Change in *Prevotella* RA, when controlled for *Pseudomonas* and *Staphylococcus*, was associated with variability in lung function change with treatment, suggesting that *Prevotella* may serve as a microbial marker of response to treatment. *Prevotella* species have been detected previously from CF sputum and bronchoalveolar lavage fluid using anaerobic culture and culture independent techniques, and suggested as potential pathogens [12], [15], [45]. Cell culture and animal lung models have found that *Prevotella intermedia* becomes cytotoxic and immunogenic when present in high quantities (>10^8^) [46]. Our data do not answer the question of whether or not *Prevotella* is a pathogen in CF; however, the number of patients in our study was small and the majority of patients were infected with *P. aeruginosa*. The three subjects with the largest change in *Prevotella* with treatment had inversely related changes in lung function. Whether these subjects represent a subgroup of patients who may benefit from antimicrobial treatment directed at *Prevotella* is not known. *Prevotella* also represent a much more heterogeneous group of organisms than *Pseudomonas*, and thus pathogenic *Prevotella* types may require more specific identification. Although we attempted to look at this using qPCR assays for specific *Prevotella* species, our small numbers limited our conclusions. Further investigation of *Prevotella* as indicator of treatment response is warranted as microbial markers could be useful in identifying underlying mechanisms of disease, identifying patients at risk for poor response and in clinical trials.

One possible explanation for the lack of association between anaerobes, inflammation and lung function, is that anaerobes may be present in sputum samples due to oral contamination from saliva. Rogers and colleagues compared bacterial communities in sputum and mouthwash from CF subjects using T-RFLP and found that oral cavity bacteria did not significantly contaminate sputum, although some bacteria were shared between the two sites [47]. Comparisons of sputum with throat swabs and bronchoalveolar lavage fluid have also indicated that anaerobes are present in the lower airways in CF [14], [16]. More recently, Goddard and colleagues called these findings into question by comparing sputum and throat samples to lung explants. They found that sputum samples overestimated the diversity and abundance of non-typical organisms compared to direct sampling of the lung, at least in patients with end-stage lung disease [48]. An alternative hypothesis for our findings is that a core microbiota including anaerobes such as *Prevotella* and *Veillonella* may be present in healthy airways with disruption of this microbiota leading to disease, as has been suggested by studies in asthma and chronic obstructive pulmonary disease [49], [50]. Given the rarity of direct lung samples, the processing, analysis and interpretation of molecular results from sputum samples is an important area for future research. Understanding the relationship between disruption of the normal microbiota and disease progression may also lead to improved treatment.

We also found that low diversity was associated with lower lung function and increased inflammation. This was driven by a predominance of *Pseudomonas* in samples with low diversity, similar to previous CF studies [18], [51]. One potential explanation is that as lung disease progresses and chronic infection develops, normal microbiota are disrupted and diversity decreases, although antibiotic use may be an important factor in this relationship [52]. Total bacterial load did not change substantially following at least one week of IV antibiotic therapy. Diversity on average did not change, although some individual subjects experienced a marked increase or decrease in diversity and effective number of species. There were also changes in RA of specific genera, particularly *Pseudomonas* and *Staphylococcus,* and decreased *P. aeruginosa* by qPCR. Anaerobic genera remained relatively stable, although individual patients experienced changes. Potential reasons for this include the use of antibiotics with limited anaerobic activity (e.g. aminoglycosides, cefipime and fluoroquinolones) in over half of subjects or resistance of anaerobes to broad-spectrum antibiotics, particularly ceftazidime and piperacillin tazobactam [16]. As in most studies of pulmonary exacerbation in CF, subjects received multiple different antibiotic combinations, limiting our ability to control for antimicrobial activity of particular antibiotics [15], [53]. The lack of change in anaerobic bacteria, however, is consistent with findings from two previous studies of CF pulmonary exacerbation, which found minimal change in the number or quantity of anaerobic organisms detected by culture and T-RFLP after treatment with IV antibiotics [15], [16]. In contrast, Daniels and Colleagues found decreased relative abundance of atypical bacterial species, including anaerobes, but an increase in *P. aeruginosa* with PEx treatment [54].

One potential limitation of molecular DNA based approaches is the inability to differentiate between live and dead bacteria [55]. It is possible that the lack of change in bacterial communities after antibiotics is due to detection of dead bacteria rather than persistence of these communities. However, bacteria targeted by typical CF antibiotics decreased, with the genera *Pseudomonas* and *Staphylococcus* both decreasing in relative abundance and *P. aeruginosa* decreasing in prevalence and quantity by qPCR. Our study also did not include sputum collections during periods of clinical stability and most early treatment sputum samples were collected after the initiation of IV antibiotics; thus, we were unable to determine whether changes occurred in bacterial communities with onset of PEx or during the first 24–72 hours of IV antibiotics. Our study, however, agrees with findings from others recently published. Tunney and colleagues collected exacerbation samples up to 48 hours after initiation of IV antibiotics, yet showed a decrease in aerobic bacteria and total viable bacteria by culture and total bacterial load by qPCR following IV antibiotic therapy [15]. Daniels and colleagues attempted to define the changes in sputum microbiota using T-RFLP analysis around the time of IV antibiotic initiation in 12 adult CF patients [54]. The degree of change was highly variable between individual patients and non- significant between day −7 days and +3 of treatment.

In conclusion, we found that early in treatment of CF PEx, *Pseudomonas* RA, but not anaerobic genera RA was associated with lower FEV_1_ and higher levels of inflammation. Furthermore, low microbial diversity was associated with higher RA of *Pseudomonas*, lower lung function and increased inflammation. With treatment, changes in RA of specific bacterial genera were markedly variable, in contrast to *Pseudomonas* which decreased. The changes in RA of anaerobic genera, with the exception of *Prevotella*, were not associated with change in FEV_1_. Given that CF PEx treatment results in variable changes of anaerobic genera suggests the need for larger studies particularly in patients without traditional CF pathogens.

## Supporting Information

Figure S1 **Schematic of 454 fusion primers (BC = bar code, L = linker).** The 454 adapter sequences are required for the sequencing platform, and the 16S primers can be targeted to any group. Our current approach targets all bacteria.
(TIF)

Figure S2 **Sequence analysis with BLAST was used to determine species specific sequences.** Species detected within the genera *Pseudomonas*, *Staphylococcus*, *Prevotella* and *Streptococcus* are shown as a proportion of total sequences for each genus.
(TIF)

Figure S3 **Scatter plot matrix showing relationships between (1) day of sample collection for early treatment, (2) FEV_1_ percent predicted, (3) Shannon diversity index (4) relative abundance of** ***Pseudomonas,*** **and (5) change in relative abundance of** ***Pseudomonas*** **with treatment.** Statistically significant correlations indicated by * (p = 0.03) and ** (p = 0.0006).
(TIF)

Figure S4 **Relative abundance of bacterial genera detected in individual sputum samples at early and late treatment.** Subject identification (SID) numbers match those in Figure 2 and Table S2.
(TIFF)

Figure S5 **Scatter plots showing the relationships between change in relative abundance of** ***Pseudomonas*** **(left column),** ***Staphylococcus*** **(middle column), and** ***Prevotella*** **(right column) and change in FEV_1_, CRP, Sputum IL-8 and Sputum NE with treatment.** Grey reference lines divide the plots into quadrants at the zero values indicating no change.
(TIF)

Figure S6 **Relationship between changes in airway microbiota and changes in lung function and inflammation with treatment (n = 16 subjects).** Results for genera present as the top ranked genus in at least one early treatment sample are displayed. Spearman’s rank correlation coefficients and 95% confidence intervals (bars) are shown, measuring the association between the changes in relative abundance of each genera with changes in FEV_1%_ predicted, C-reactive protein (CRP) and sputum neutrophil elastase (NE).
(TIF)

Figure S7 **Quantitative culture results for** ***Pseudomonas aeruginosa*** **and** ***Staphylococcus aureus*** **for samples positive and negative for** ***Pseudomonas*** **and** ***Staphylococcus*** **by pyrosequencing.**
(TIF)

Table S1 **Primers used for qPCR specific organism assays.**
(XLSX)

Table S2 **Subject microbiologic and clinical data.**
(XLSX)

Table S3 **Microbiota detected from all sputum samples (early and late treatment). Genera present in greater than 1% relative abundance are listed.** N = 37 sputum samples.
(XLSX)

Table S4 **Results of total and specific bacterial qPCR assays at early and late treatment, and median change with IV antibiotic treatment.**
(XLSX)

Table S5 **Association between total and specific bacteria quantified by qPCR assays and FEV_1_, age and inflammatory markers at early treatment (n = 21 sputum samples).** P-values <0.05 are highlighted.
(XLSX)

Table S6 **Model results associating the change in relative abundance of three microbial markers with response to treatment measured by change in FEV1% predicted.**
(XLSX)

Data S1 **Methods and Results.**
(DOCX)
