# Supplementary figures and images for: Inflammation and Airway Microbiota during Cystic Fibrosis Pulmonary Exacerbations
Source: PLoS One. 2013 Apr 30;8(4):e62917. doi: 10.1371/journal.pone.0062917 (PMC3639911; doi:10.1371/journal.pone.0062917)

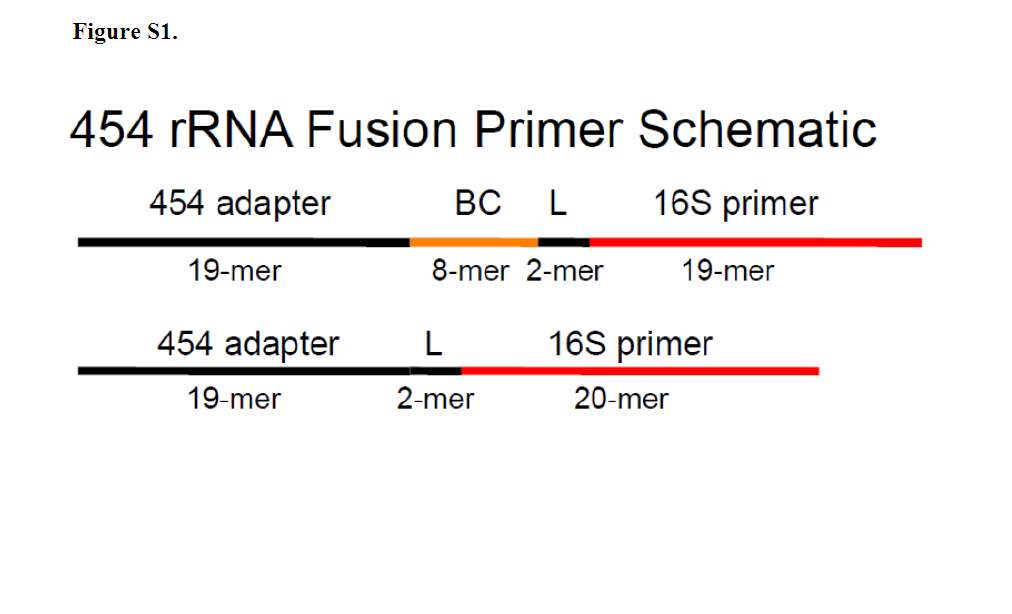

Supplement: Figure S1 — Schematic of 454 fusion primers (BC = bar code, L = linker). The 454 adapter sequences are required for the sequencing platform, and the 16S primers can be targeted to any group. Our current approach targets all bacteria. (TIF) [file pone.0062917.s001.tif]

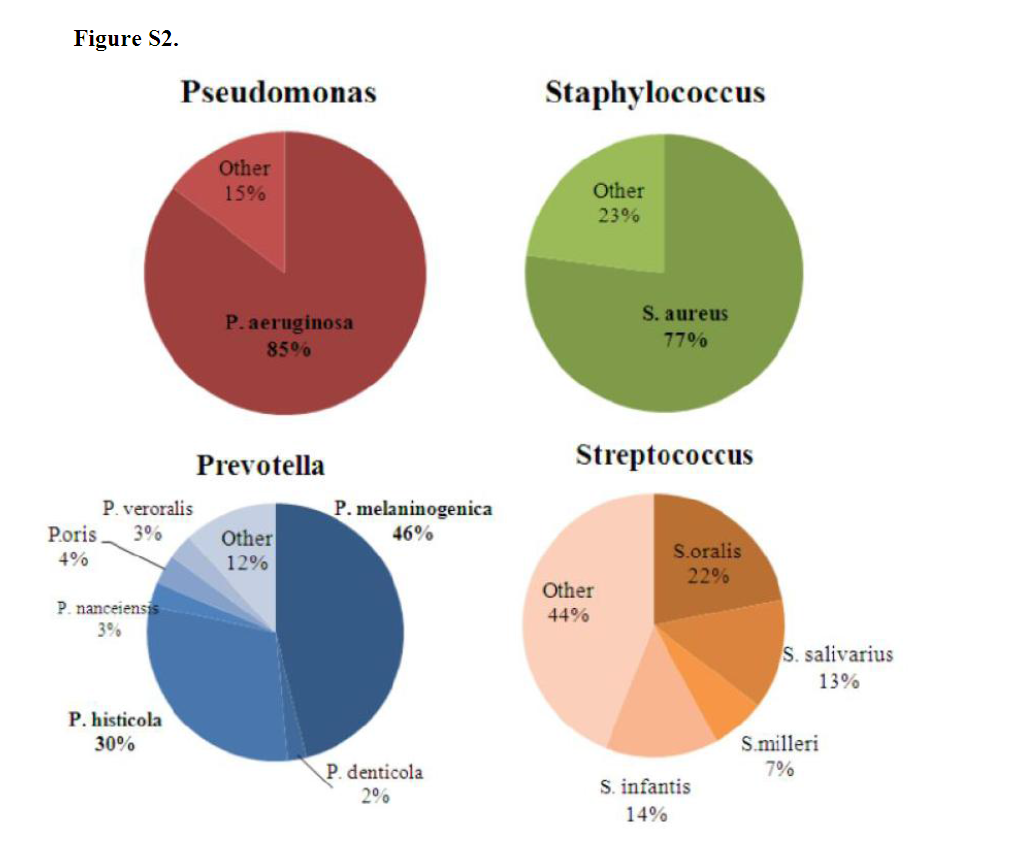

Supplement: Figure S2 — Sequence analysis with BLAST was used to determine species specific sequences. Species detected within the genera Pseudomonas, Staphylococcus, Prevotella and Streptococcus are shown as a proportion of total sequences for each genus. (TIF) [file pone.0062917.s002.tif]

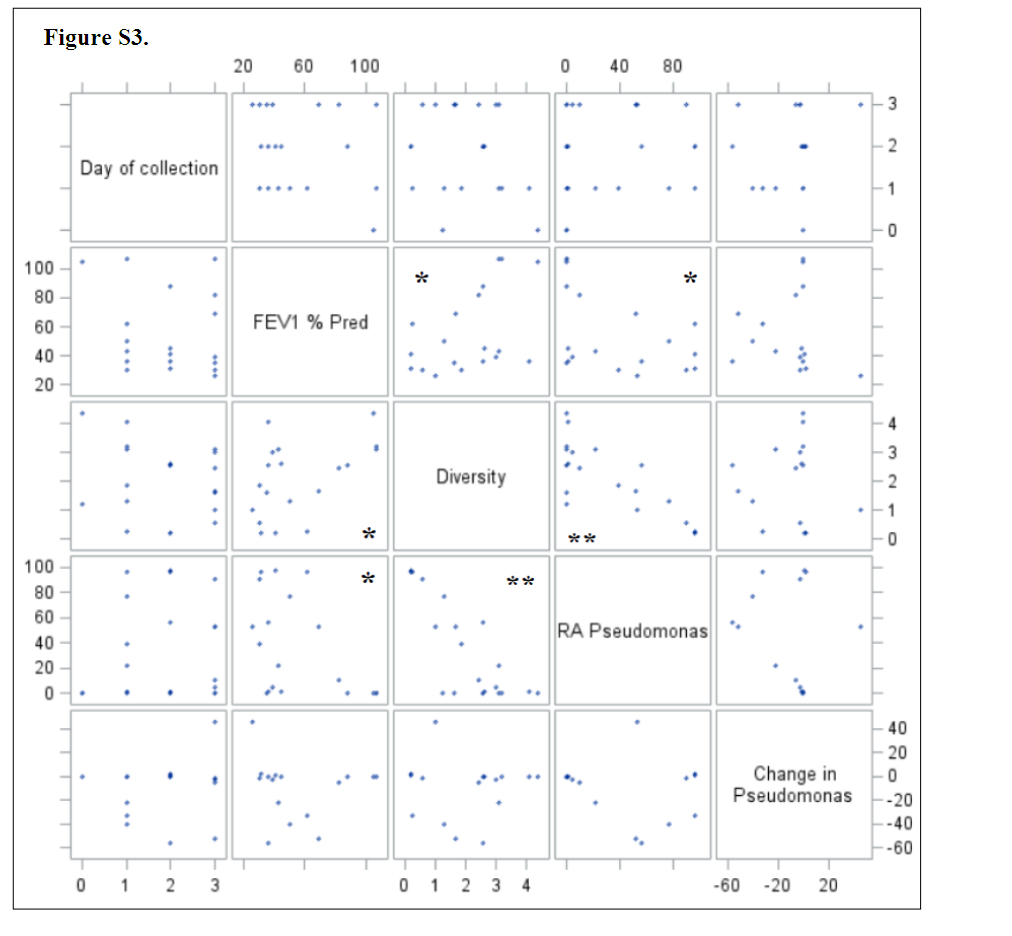

Supplement: Figure S3 — Scatter plot matrix showing relationships between (1) day of sample collection for early treatment, (2) FEV1 percent predicted, (3) Shannon diversity index (4) relative abundance of Pseudomonas, and (5) change in relative abundance of Pseudomonas with treatment. Statistically significant correlations indicated by * (p = 0.03) and ** (p = 0.0006). (TIF) [file pone.0062917.s003.tif]

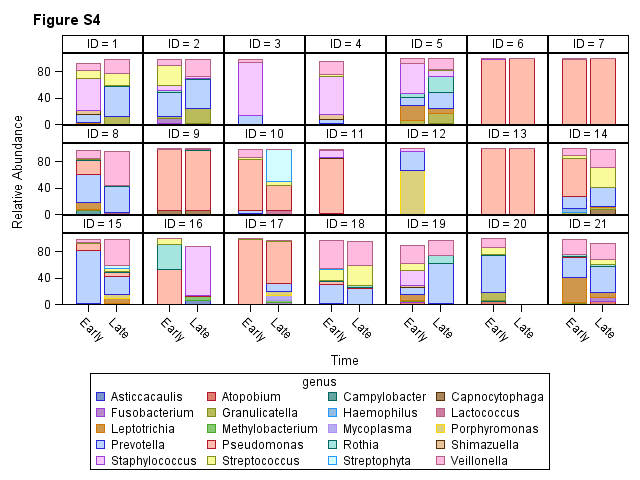

Supplement: Figure S4 — Relative abundance of bacterial genera detected in individual sputum samples at early and late treatment. Subject identification (SID) numbers match those in Figure 2 and Table S2. (TIFF) [file pone.0062917.s004.tif]

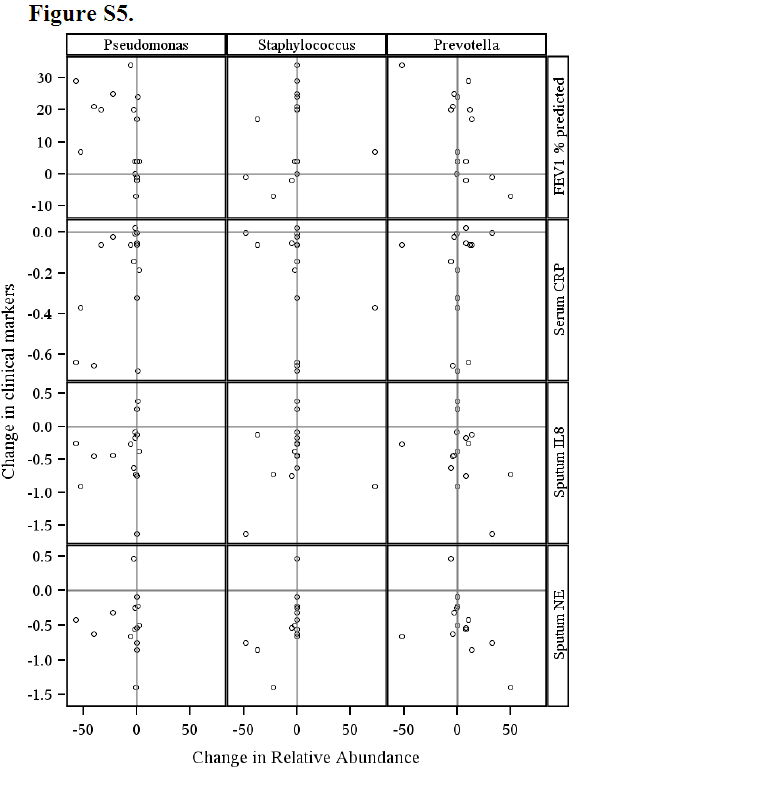

Supplement: Figure S5 — Scatter plots showing the relationships between change in relative abundance of Pseudomonas (left column), Staphylococcus (middle column), and Prevotella (right column) and change in FEV1, CRP, Sputum IL-8 and Sputum NE with treatment. Grey reference lines divide the plots into quadrants at the zero values indicating no change. (TIF) [file pone.0062917.s005.tif]

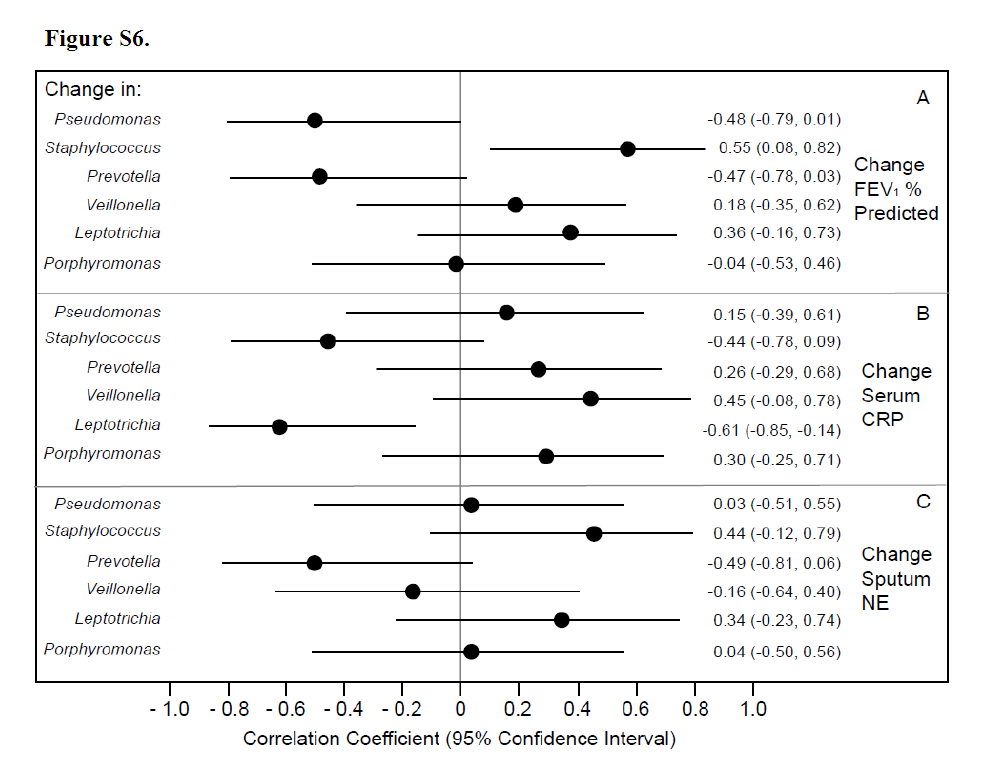

Supplement: Figure S6 — Relationship between changes in airway microbiota and changes in lung function and inflammation with treatment (n = 16 subjects). Results for genera present as the top ranked genus in at least one early treatment sample are displayed. Spearman’s rank correlation coefficients and 95% confidence intervals (bars) are shown, measuring the association between the changes in relative abundance of each genera with changes in FEV1% predicted, C-reactive protein (CRP) and sputum neutrophil elastase (NE). (TIF) [file pone.0062917.s006.tif]

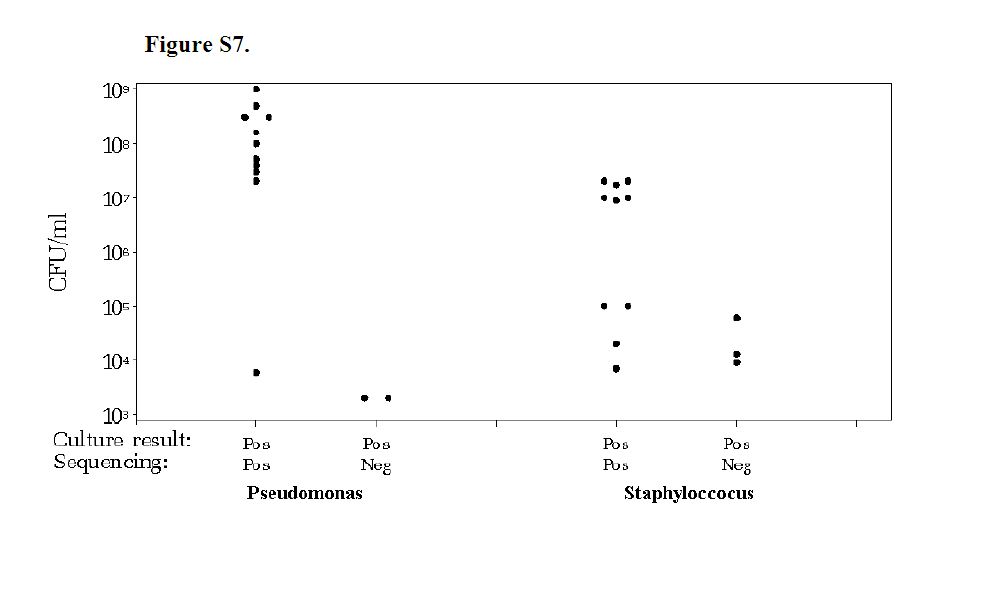

Supplement: Figure S7 — Quantitative culture results for Pseudomonas aeruginosa and Staphylococcus aureus for samples positive and negative for Pseudomonas and Staphylococcus by pyrosequencing. (TIF) [file pone.0062917.s007.tif]
