# Supplementary material for: Inflammation and Airway Microbiota during Cystic Fibrosis Pulmonary Exacerbations
Source: PLoS One. 2013 Apr 30;8(4):e62917. doi: 10.1371/journal.pone.0062917 (PMC3639911; doi:10.1371/journal.pone.0062917)
Supplement: Data S1 — Methods and Results. (DOCX) [file pone.0062917.s014.docx]

**Online Data Supplement**

**Inflammation and airway microbiota during cystic fibrosis pulmonary exacerbations**

Authors: Edith T. Zemanick^1^, J. Kirk Harris^1^, Brandie D. Wagner^2^, Charles E. Robertson^3^, Scott D. Sagel^1^, Mark J. Stevens^1^, Frank J. Accurso^1^ and Theresa A. Laguna^4^

^1^ Department of Pediatrics, University of Colorado School of Medicine, Aurora, Colorado

^2^ Department of Biostatistics and Informatics, Colorado School of Public Health, University of Colorado School of Medicine, Aurora, Colorado

^3^ Department of Molecular, Cellular and Developmental Biology, University of Colorado, Boulder, Colorado

^4^ Department of Pediatrics, University of Minnesota Medical School and the University of Minnesota Amplatz Children’s Hospital, Minneapolis, MN

**METHODS**

**Sputum collection, processing and culture:** Spontaneously expectorated sputum was collected and split into two sterile containers. One container was submitted to the clinical microbiology laboratory for quantitative microbiology according to consensus guidelines for CF respiratory cultures.[1] The second container was sent to the Clinical Translational Research Center (CTRC) Core Laboratory for molecular microbiologic studies and measurement of biomarkers of inflammation. In the CTRC Core Laboratory, sputum was processed and homogenized within 20 minutes. Sputum weight was determined and three parts sterile room temperature Phosphate-buffered saline (Gibco BRL, Grand Island, NY) and four parts volume of sterile 10% Dithiothreitol (Caldon Biotech, Vista, CA) added. Samples less than 0.5 grams were deemed inadequate and further processing was not done. The samples were then incubated in a shaking water bath at 37ºC for 15 minutes, and gently mixed using a transfer pipette at 5-minute intervals. If the sample was not completely homogenized at 15 minutes, it was incubated again in the 37ºC shaking water bath for 5-10 minutes. Following homogenization, 1.1 ml was removed and mixed with Trypan Blue stain for cell count using a standard hemacytometer and Wright stain for differential cell count. An additional 2 ml of homogenized sputum was removed and frozen at -70 ºC for molecular microbiologic studies as described below.

**Sputum processing for inflammatory biomarkers**: The remaining homogenized sputum sample was centrifuged at 500 x *g* for 20 minutes at 4 ºC. The supernatant was aspirated and centrifuged a second time at 4,000 x g for 20 minutes. The supernatants from the second spin were divided into two aliquots. One aliquot was treated with protease inhibitors, phenylmethylsulfonylfluoride (PMSF) (Sigma Diagnostics, St. Louis, MO) and ethylenediamenetetraacetic acid (EDTA) (Sigma Diagnostics, St. Louis, MO). The second aliquot was left untreated. Both aliquots were frozen at -70 ºC for later analyses. Protease inhibitor treated supernatant was used for interleukin-8 (IL-8) assay and untreated supernatant used for free neutrophil elastase (NE) assay. These procedures have been previously described for analysis of inflammatory biomarkers.[2]

**Quantitative PCR**

Bacterial load assay**:** Total ribosomal RNA gene copy number was measured using a quantitative real-time PCR (qPCR) TaqMAN assay (Roche Molecular Systems, Inc.) that has been published previously.[3,4] Primers used were the forward primer, 5'-TCCTACGGGAGGCAGCAGT-3', the reverse primer, 5'-GGACTACCAGGGTATCTAATCCTGTT-3' and the probe, (6-FAM)-5'-CGTATTACCGCGGCTGCTGGCAC-3'-(TAMRA). A cloned bacterial rRNA gene (*P. melaninogenica*) was used as the standard ranging in dilution from 10^2^ to 10^8^ copies on each plate. Samples were run in triplicate and mean values calculated. The PCR reaction was performed in a total volume of 25ul using the TaqMan Universal PCR Master Mix (ABI), containing 100nM of each of the universal forward and reverse primers and the fluorogenic probe. The reaction conditions for amplification of DNA were 50ºC for 2 minutes, 95ºC for 10 minutes and 40 cycles of 95ºC for 15 s and 60ºC for 1 minute. Resulting values were log_10_ transformed.

Specific organism assays: Ribosomal RNA gene copy numbers for *Pseudomonas aeruginosa, Prevotella denticola, Prevotella melaninogenica* and *Prevotella oris* were measured using specific organism qPCR assays, which have been published previously.[4–6] The qPCR reactions were run using the Power SYBR Green PCR Master Mix (Applied Biosystems, Carlsbad, CA) and the appropriate PCR primers. (Table S1) Bacterial standards ranging from 10^2^ to 10^8^ copies were run on each plate. Melting temperatures were measured for each qPCR reaction and values outside the pre-specified range for each organism were considered not detected. Samples were run in triplicate; resulting values were log_10_ transformed and mean values calculated. PCR conditions varied by assay and are described below. *P. aeruginosa* amplification consisted of a three step program of 94^o^C for 20s, 60^o^C for 20s and 72^o^C for 60s. The *Prevotella* assays used a two-step program of 95^o^C for 15s and 60^o^C for 60s. Assays were run with 35-40 cycles.

**RESULTS**

**Comparison of pyrosequencing and qPCR to culture for detection of CF pathogens**

*Pseudomonas* was detected by pyrosequencing in 12 of 14 sputum samples that were positive by culture (86%) and in 2 of 7 samples that were negative by culture. In the two culture negative samples, *Pseudomonas* sequences accounted for <1% of total sequences, compared to 4-99% of sequences in those with positive cultures. Using BLAST analysis, *Pseudomonas* was identified as *P. aeruginosa* and *P.* *fluorescens* from the two negative culture samples, respectively. One additional sample that was negative by culture was identified as having *P. aeruginosa* by BLAST analysis (<1% RA) but not by RDP classification. QPCR detected *P. aeruginosa* in all but one of the 14 admission sputum samples with a positive culture (93% sensitivity); the discrepancy was not in the same sample as for sequencing. QPCR detected *P. aeruginosa* in 4 samples that were negative by culture. Quantification ranged from 6.1-7.5 log_10_ gene copies/mL in those with a negative culture, compared to 6.7-10.5 log_10_ gene copies/mL in those samples with a positive culture. The one sample with positive culture but negative qPCR had 10^7^ cfu/ml *P. aeruginosa* by culture and 97% of sequences identified as *P. aeruginosa*, suggesting a failure of the assay rather than presence of bacteria below the limit of detection.

*Staphylococcus* was detected by pyrosequencing in 10 of 13 specimens positive by culture (77%); no culture negative samples were positive by sequencing. *Haemophilus* was detected in low abundance in two samples by pyrosequencing; using BLAST data, *Haemophilus* from one sample was identified as *H. influenzae* (RA 5%) and as *H. parainfluenzae* (RA <1%) in the other sample. No specimens were culture positive for *H. influenzae*. *Stenotrophomonas* was detected by pyrosequencing in one culture negative sample (confirmed as *S. maltophilia* by BLAST) but in very low abundance (0.1%); conversely one sample was positive by culture but negative by pyrosequencing. Quantitative culture results for samples based on pyrosequencing results are shown in Figure S6.

Online Supplement References

1. Burns JL, Emerson J, Stapp JR, Yim DL, Krzewinski J, Louden L, Ramsey BW, Clausen CR (1998) Microbiology of sputum from patients at cystic fibrosis centers in the United States. Clin Infect Dis 27: 158-163.

2. Sagel SD, Kapsner R, Osberg I, Sontag MK, Accurso FJ (2001) Airway inflammation in children with cystic fibrosis and healthy children assessed by sputum induction. Am J Respir Crit Care Med 164: 1425-1431.

3. Nadkarni MA, Martin FE, Jacques NA, Hunter N (2002) Determination of bacterial load by real-time PCR using a broad-range (universal) probe and primers set. Microbiology 148: 257-266.

4. Zemanick ET, Wagner BD, Sagel SD, Stevens MJ, Accurso FJ, Harris JK (2010) Reliability of quantitative real-time PCR for bacterial detection in cystic fibrosis airway specimens. PLoS ONE 5: e15101. 10.1371/journal.pone.0015101 [doi].

5. Nadkarni MA, Caldon CE, Chhour KL, Fisher IP, Martin FE, Jacques NA, Hunter N (2004) Carious dentine provides a habitat for a complex array of novel *Prevotella*-like bacteria. J Clin Microbiol 42: 5238-5244.

6. Matsuda K, Tsuji H, Asahara T, Kado Y, Nomoto K (2007) Sensitive quantitative detection of commensal bacteria by rRNA-targeted reverse transcription-PCR. Appl Environ Microbiol 73: 32-39.
